# Screening and diagnostic tools for autism spectrum disorder: Systematic review and meta-analysis

**DOI:** 10.1016/j.clinsp.2023.100323

**Published:** 2024-03-14

**Authors:** Clara Lucato dos Santos, Indyanara Inacio Barreto, Idevaldo Floriano, Luca Schiliró Tristão, Antonio Silvinato, Wanderley Marques Bernardo

**Affiliations:** a Departamento de Medicina Baseada em Evidências, Faculdade de Ciências Médicas de Santos (UNILUS), Santos, SP, Brazil; b Núcleo de ATS da Unimed de Campinas, Campinas, SP, Brazil; c Medicina Baseada em Evidências, Cooperativa Baixa Mogiana, Mogi-Guaçu, SP, Brazil; d Medicina Baseada em Evidências, Associação Médica Brasileira, São Paulo, SP, Brazil; e Faculdade de Medicina, Universidade de São Paulo, São Paulo, SP, Brazil

**Keywords:** Autism, Children, Screening, Diagnosis

## Abstract

**Introduction:**

Autism Spectrum Disorder (ASD) is a heterogeneous neurodevelopmental disorder, with main manifestations related to communication, social interaction, and behavioral patterns. The slight dynamics of change in the child over time require that the onset of clinical manifestations presented by the child be more valued, with the aim of stabilizing the condition. Faced with a variety of methods for diagnosing ASD, the question arises as to which method should be used. This systematic review aims to recommend the best tools to perform screening and diagnosis.

**Methodology:**

This systematic review followed the PRISMA guidelines. The databases MEDLINE, Embase, CENTRAL (Cochrane), and Lilacs were accessed, and gray and manual searches were performed. The search strategy was created with terms referring to autism and the diagnosis/broad filter. The studies were qualitatively evaluated and quantitatively. Statistical analysis was performed using Meta-diSc-2.0 software, the confidence interval was 95 %.

**Results:**

The M-CHAT-R/F tool demonstrated a sensitivity of 78 % (95 % CI 0.57‒0.91) and specificity of 0.98 (95 % CI 0.88–1.00). The diagnostic tools demonstrated sensitivity and specificity respectively of: ADOS, sensitivity of 87 % (95 % CI 0.79‒0.92) and specificity 75 % (95 % CI 0.73‒0.78); ADI-R demonstrated test sensitivity of 77 % (95 % CI 0.56‒0.90) and specificity 68 % (95 % CI 0.52‒0.81), CARS test sensitivity was 89 % (95 % CI 0.78‒0.95) and specificity 79 % (95 % CI 0.65‒0.88).

**Conclusion:**

It is mandatory to apply a screening test, the most recommended being the M-CHAT-R/F. For diagnosis CARS and ADOS are the most recommended tools.

## Highlights

• Autism Spectrum Disorder (ASD) is a heterogeneous neurodevelopmental disorder.
• Autism spectrum disorder has a significant impact on the patient and their family.
• To provide adequate advice is to carry out screening and diagnosis correctly and accurately.
• Screening test must be applied, M-CHAT-R/F is recommended.
• For diagnosis CARS and ADOS are the most recommended tools.

## Introduction

Autism Spectrum Disorder (ASD) constitutes a multifaceted neurodevelopmental spectrum, encompassing a diverse array of conditions including autistic disorder, Asperger's Disorder, childhood disintegrative disorder, and pervasive developmental disorders, as described by the DSM-V.^1^ Main characteristics are deficits in communication, social interaction, and repetitive behaviors,^2^ ASD presents a compelling challenge in both clinical and social contexts.

The global prevalence of ASD, averaging 65 cases per 10,000 individuals, marks it as a considerable public health concern. This prevalence notably varied across periods, reflecting the profound impact of evolving diagnostic paradigms and methodologies on disease frequency.^3^

The slight dynamic of change in the child over time requires that the onset of clinical manifestations presented by the child be more valued, with the aim of stabilizing the condition. Early diagnosis, carried out by a multidisciplinary team,^4^ means that early approaches are adopted, impacting the patient's prognosis and integration into society.^5^, ^6^, ^7^, ^8^

Little is known about the complex pathophysiology of ASD, making it more difficult to make an explanatory diagnosis. In view of this, it must be understood that the diagnosis of autism spectrum disorder constitutes a description and not an explanation.^4^

However, among the ASD's pathophysiological complexities, the diagnostic process remains predominantly descriptive rather than explanatory. Comprehensive assessments conducted by healthcare professionals aim to unravel the nature of patient challenges, encompassing functional and nosological dimensions.^4^ ASD diagnosis includes impairments in communication, social interaction, and behavior, creating a multidimensional diagnostic landscape.

Distinguishing between medical diagnosis and behavioral assessments across varied spheres underscores the intricate nature of diagnosing ASD, a condition manifesting diversely without a stringent, universally applicable accuracy standard.^9^^,^^10^

In order to make an early diagnosis, screening tools are commonly applied in medical practice with the intention of carrying out risk screening among the entire population. The American Academy of Pediatrics suggests that routine screening assessment for ASD be carried out at a consultation between 18 and 24 months,^11^ however it can be carried out at other stages of life. Screening tools were developed with the aim of identifying symptoms early and promoting greater surveillance of children at high risk of developing them.^2^

The most commonly used screening tool is the M-CHAT, a two-step assessment that includes a 23-item parent questionnaire and a follow-up interview for some cases with the aim of impacting the number of false positive cases. The Modified Checklist for Autism in Children, Revised, with Follow-up (M-CHAT–R/F) has the same objective as the M-CHAT, but it has been reformulated, some items have been removed and new scoring criteria based on follow-up have been adopted.^12^^,^^13^

Once it is determined that a child is at risk for an ASD diagnosis, whether through screening or surveillance, a timely referral for clinical diagnostic evaluation and early intervention or school-based services is indicated, depending on their age.^14^

Among the most used tools to evaluate the diagnosis of autism are the DSM-V criteria, the ADI-R and ADOS questionnaire. These last two have undergone revisions over time and have undergone small modifications according to their edition with the aim of becoming more accurate.

Autism spectrum disorder has a significant impact on the patient and their family, which requires specialized attention and efforts so that they are increasingly integrated into society. Economic and social detachments are necessary so that there is minimum accessibility to rights. The first step to providing adequate advice is to carry out screening and diagnosis correctly and accurately. Therefore, high-quality evidence must be used to find tools to make the diagnosis in the best possible way.

## Method

Preferred Reporting Items for a Systematic Review and Meta-analysis of Diagnostic Test Accuracy Studies (PRISMA-DTA) guidelines^15^ and details are registered in the International Prospective Register of Systematic Reviews (PROSPERO).^16^

The MEDLINE, Embase, Cochrane Central and Lilacs databases were evaluated, in addition to manual searches. The search was carried out between March and August 2023. Terms were searched in titles, abstract and keywords.

The search strategy used was:

• MEDLINE (PubMed): (Autism Spectrum Disorder OR Autism Spectrum Disorders OR Autistic Disorder OR Autism) AND Diagnosis/broad[filter].
• Embase: (Autism Spectrum Disorder OR Autism Spectrum Disorders OR Autistic Disorder OR Autism) AND (Diagnosis).
• LILACS: (Autism Spectrum Disorder OR Autism Spectrum Disorders OR Autistic Disorder OR Autism) AND (Diagnosis).
• CENTRAL (Cochrane): (Autism Spectrum Disorder OR Autism Spectrum Disorders OR Autistic Disorder OR Autism) AND (Diagnosis).

Filter for children and adolescents were adopted in MEDLINE, Embase and Lilacs.

The eligibility criteria for the studies were: (I) Age <18-years; (II) Screening and/or diagnosis assessment through questionnaires (III) Cross-sectional studies; (IV) Without period restrictions; (VI) Without language restrictions; and (VII) Full text or summary with relevant data is available.

The titles and abstracts identified in the search were evaluated by two authors independently, and those that met the inclusion criteria were selected for review. In cases of disagreement, a third author was consulted.

The following data were extracted from the selected studies: name, year of publication, population, questionnaire description, sensitivity, specificity, true positive, true negative, false positive, false negative and prevalence.

The risk of bias will be accessed using the QUADAS-2 tool^17^ and classified as low, medium and high.

### Data analysis

Data analysis was performed using extracted data, a 2 × 2 contingency table was constructed for each study, which included the number of true positives (VPs), False Positives (FPs), False Negatives (FNs), and True Negatives (TNs). The main measures of diagnostic accuracy were sensitivity and specificity.

The Meta-DiSc software version 2.0 (Clinical Biostatistics Unit of the Ramón y Cajal Research Institute, Madrid, Spain) was used for meta-analyses of Diagnostic Test Accuracy (ATD) studies.^18^ Meta-DiSc 2.0 performed statistical analyses using a bivariate random effects model or a univariate random effects model for meta-analyses with 3 or fewer studies. Pooled accuracy estimates, including sensitivity and specificity, positive and negative predictive likelihood ratios, diagnostic Odds Ratio, and false positive rate along with their 95 % Confidence Intervals (95 % CIs) were calculated. Forest plots and Summary Receiver Operating Characteristic (SROC) curves were created by the software. Heterogeneity was assessed using logit variances of sensitivity and specificity, bivariate I2 index, area of the 95 % prediction ellipse, and median odds ratios for sensitivity and specificity.^18^

## Results

A total of 10617 articles were accessed after removing duplicates. Of these, 215 titles and abstracts were selected for eligibility criteria assessment, of which 90 were selected for full-text analysis. Finally, 19 articles were included in the meta-analysis (Fig. 11 Appendices – Flow diagram) (Supplementary Table 1)

The selected studies have samples ranging from 40 to 11876 patients in the screening analysis and 45 to 1039 patients in the diagnostic analysis. The age range of the children ranges from 11-months to 18-years (Supplementary Tables 4 and 5). Sensitivity, specificity, and likelihood ratio analyses were performed for screening and diagnostic tests. Eleven studies presented a moderate risk of bias, while eight presented a low risk (QUADAS-2)^17^ (Supplementary Tables 1, 2 and 3).

### Screening CE

Six studies^19^, ^20^, ^21^, ^22^, ^23^, ^24^ evaluated the M-CHAT-R/F tool for screening. The test was applied to 10,756 children, with positive results in 168 patients. The prevalence was 2 %. The sensitivity of the methods was 78 % and specificity 98 %. The positive likelihood ratio is 35.62 (95 % CI 6.19–205.07) and the negative likelihood ratio is 0.225 (95 % CI 0.10–0.48).

**Fig. 1:**
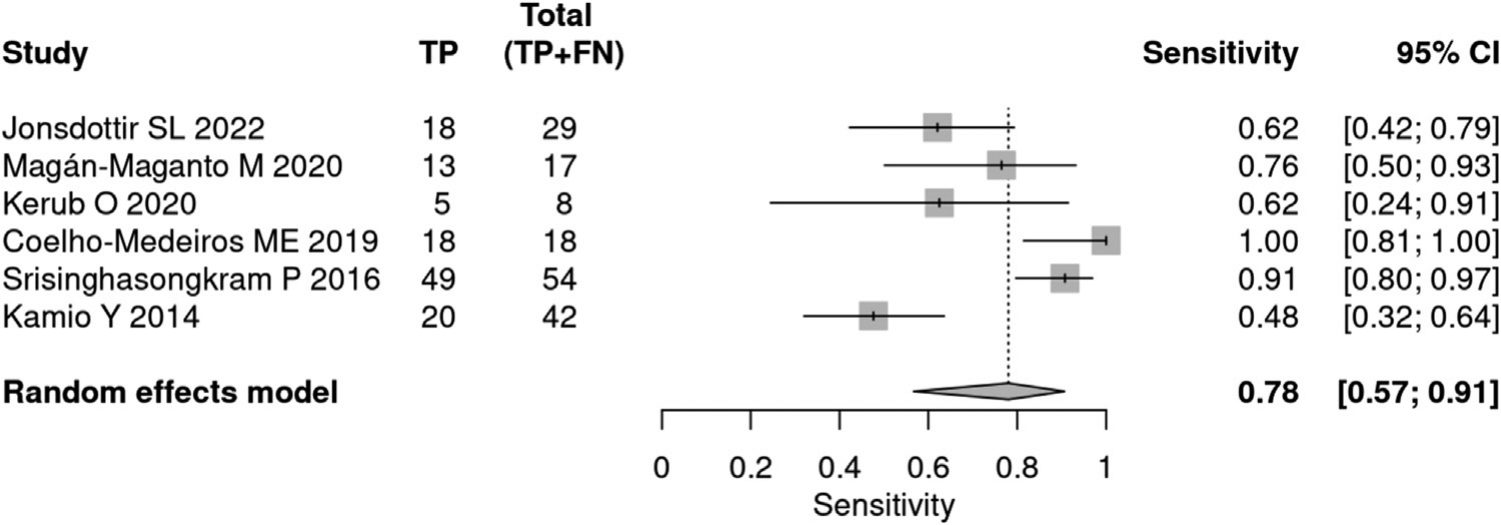
Forest plot sensitivity – screening test.

**Fig. 2:**
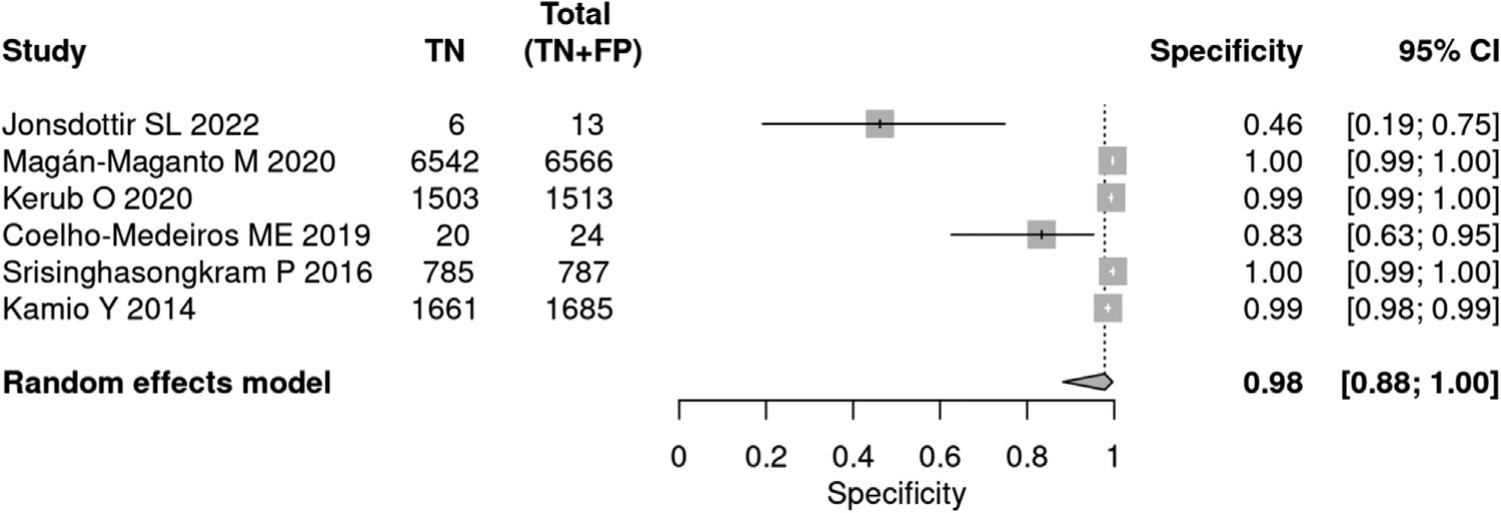
Forest plot specificity – screening test.

The post-test probability in a 50 % prevalence context was 97 %. When the prevalence was 2 %, the post-test probability was 42 % (Fig. 1, Fig. 2).

### Diagnosis

#### Global analysis

**Fig. 3:**
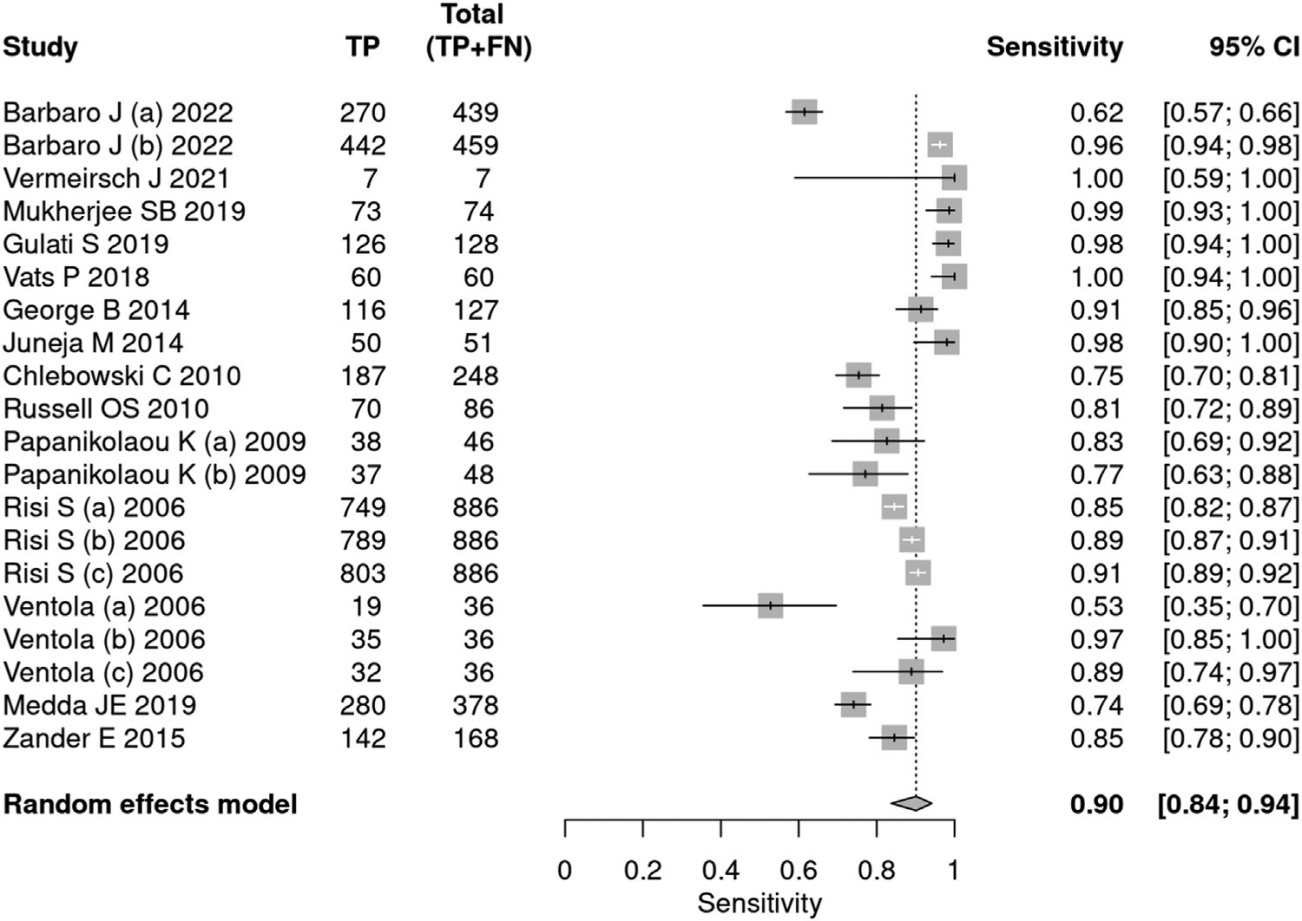
Forest plot sensitivity – diagnostic tests.

**Fig. 4:**
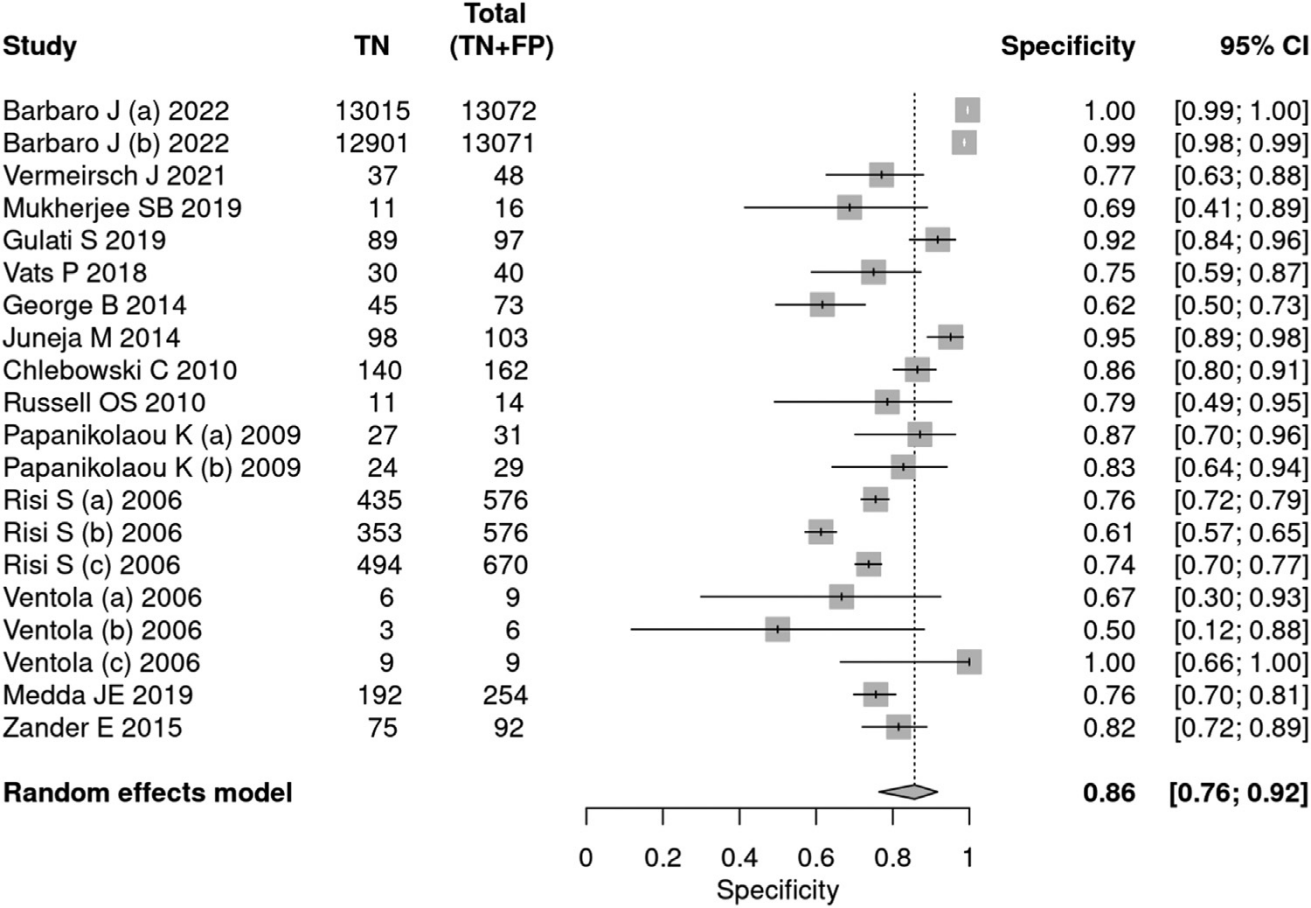
Forest plot specificity – diagnostic tests.

Fourteen articles^25^, ^26^, ^27^, ^28^, ^29^, ^30^, ^31^, ^32^, ^33^, ^34^, ^35^, ^36^, ^37^, ^38^ used tools to diagnose autism, including ADOS, ADI-R, CARS, and SARS. A total of 34,003 patients were evaluated, of which 5,085 received a positive test result, indicating a prevalence of 15 %. The sensitivity of the methods was 90 % and specificity 86 %. The positive likelihood ratio was 6.294 (95 % CI 3.742–10.587) and the negative likelihood ratio was 0.116 (95 % CI 0.07–0.191) (Fig. 3, Fig. 4).

#### ADOS

Six articles^26^^,^^31^^,^^36^, ^37^, ^38^, ^39^ applied the ADOS tool for the diagnosis of autism. A total of 2622 patients were evaluated, of which 1521 had a positive test result, indicating a prevalence of 58 %. The sensitivity of the test was 87 % and the specificity 75 %. The positive likelihood ratio was 3.520 (95 % CI 3.163–3.919) and the negative likelihood ratio was 0.174 (95 % CI 0.107–0.283).

**Fig. 5:**
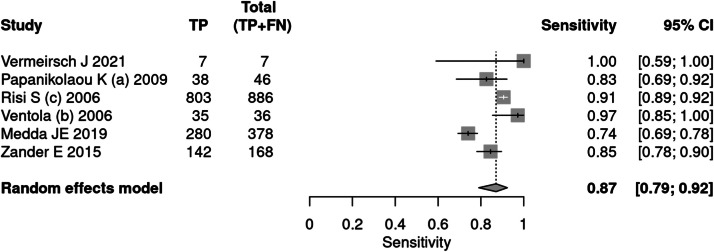
Forest plot sensitivity ‒ ADOS test.

**Fig. 6:**
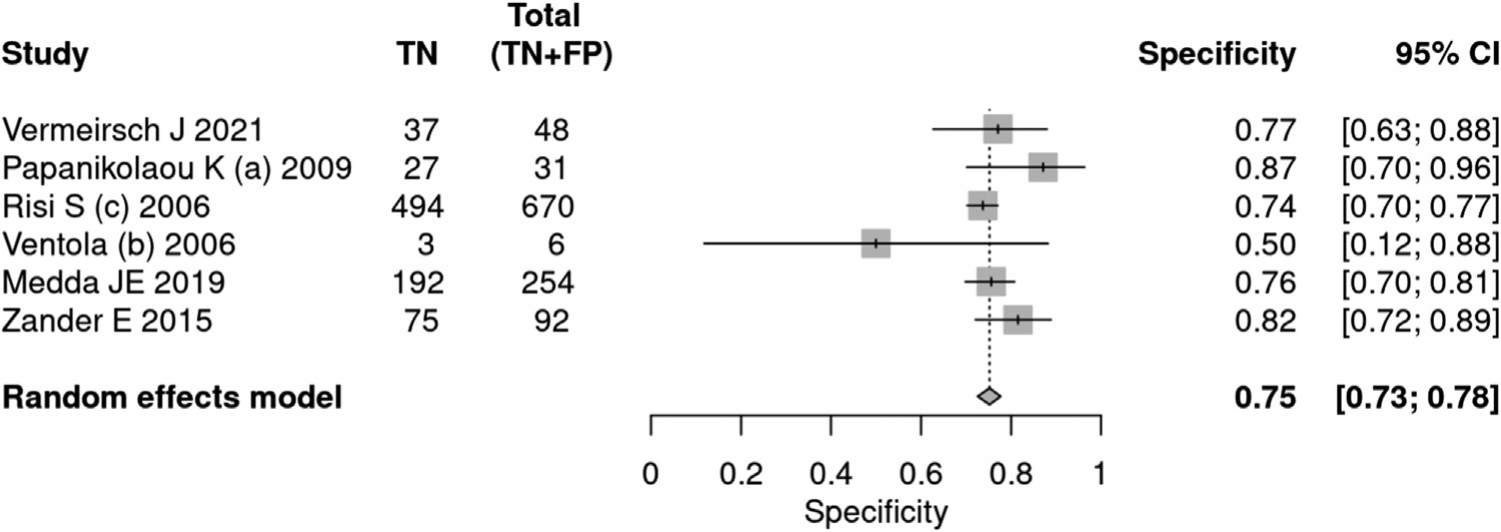
Forest plot specificity ‒ ADOS test.

In a 50 % prevalence scenario, the post-test probability was 77 %. When the prevalence considered was 15 %, the post-test probability was 38 %. After screening, in a context of prevalence of 42 %, the post-test probability was 71 % (Fig. 5, Fig. 6).

#### ADI-R

Three articles^36^, ^37^, ^38^ used the ADI-R tool as an instrument for diagnosing autism. A total of 1584 patients were evaluated, of which 970 received a positive test result, indicating a prevalence of 61 %. The sensitivity of the test was 77 % and specificity 68 %. The positive likelihood ratio was 2.401 (95 % CI 1.445–3.99) and the negative likelihood ratio was 0.34 (95 % CI 0.16–0.723).

**Fig. 7:**
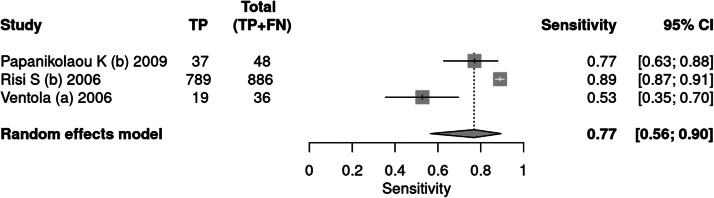
Forest plot sensitivity ‒ ADI-R test.

**Fig. 8:**
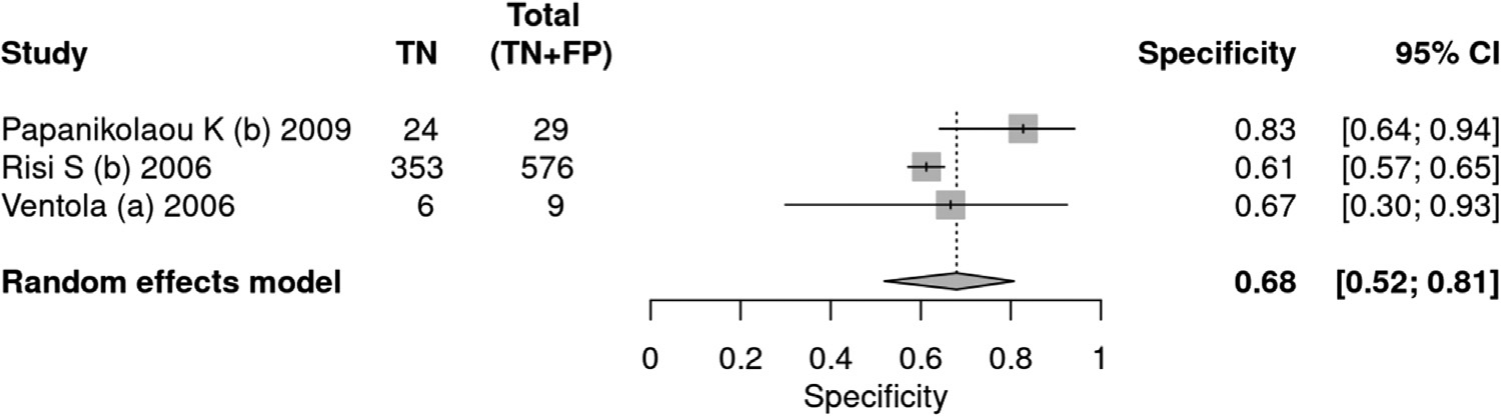
Forest plot specificity ‒ ADI-R test.

In a 50 % prevalence scenario, the post-test probability was 70 %. When the prevalence considered was 15 %, the post-test probability was 29 %. After screening, in a context of prevalence of 42 %, the post-test probability was 63 % (Fig. 7, Fig. 8).

#### CARS

Five articles^27^^,^^32^^,^^35^^,^^38^^,^^34^ applied the CARS tool for the diagnosis of autism. A total of 845 patients were evaluated, of which 571 received a positive test result, indicating a prevalence of 68 %. The sensitivity of the test was 89 % and specificity 79 %. The positive likelihood ratio was 3.637 (95 % CI 2.461–5.374) and the negative likelihood ratio was 0.156 (95 % CI 0.092–0.263).

**Fig. 9:**
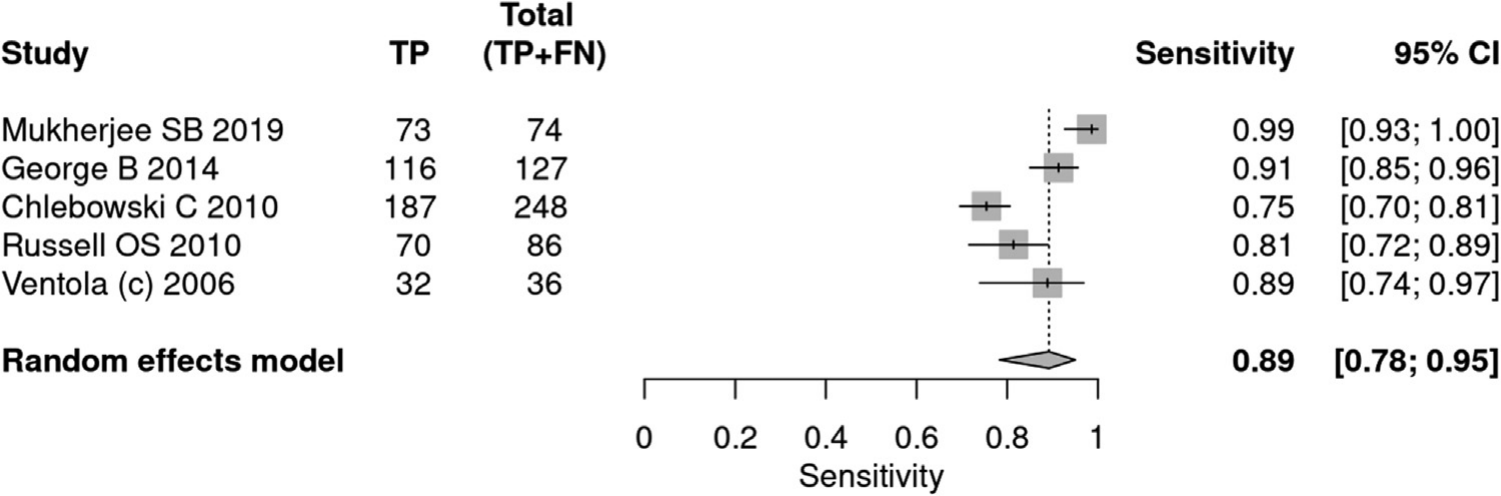
Forest plot sensitivity ‒ CARS test.

**Fig. 10:**
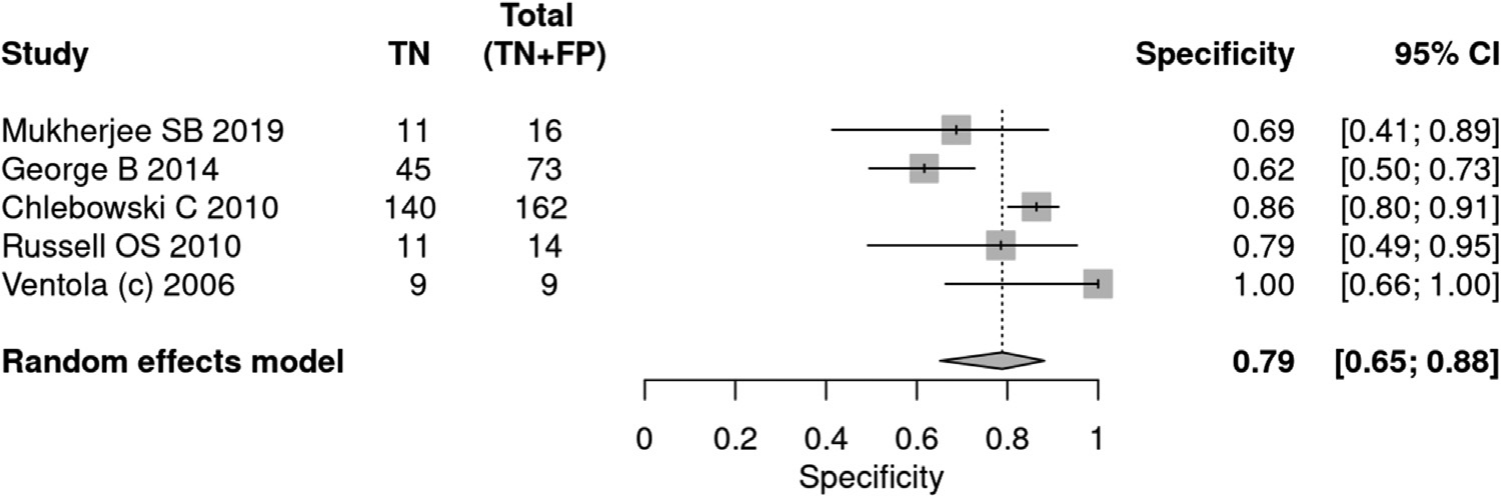
Forest plot specificity ‒ CARS test.

In a 50 % prevalence scenario, the post-test probability was 78 %. When the prevalence considered was 15 %, the post-test probability was 38 %. After screening, in a context of prevalence of 42 %, the post-test probability was 64 % (Fig. 9, Fig. 10).

## Discussion

This systematic review and meta-analysis demonstrated, based on the post-test probability, that the chance of an individual being affected and identified with ASD in a context of prevalence of 50 %, according to the ADOS, ADI-R, and CARS diagnostic tests, is 77 %, 70 %, 78 %, respectively.

Given the diversity of screening and diagnostic tools, it is extremely important to understand their characteristics and evaluated criteria. Among the tools available for screening, M-CHAT-R/F is the most used. For ADOS diagnosis, ADI-R and CARS are the most used. Their descriptions can be seen in Supplementary File 1.

In a scenario of uncertain ASD diagnosis, studies lacking robust design, comparative analysis, imprecise methodologies, and population delimitations underscore the challenge of accurate diagnostic outcomes. This situation often leads to screening errors and imprecise diagnoses due to varying sensitivities and specificities among tools. This challenge underlines the need for highly sensitive and specific methods tailored to specific age groups for effective ASD screening and diagnosis.

### Limitations

While the present findings provide relevance, caution is warranted in their careful interpretation. The high heterogeneity in methodologies across studies might have influenced the results. Patient selection for diagnostic instrument evaluation often occurred within communities where the screening test was previously applied.

Most of the studies retrieved had a case-control study design, unsuitable for a comprehensive analysis of screening and diagnostic tools, as the ideal method involves cross-sectional studies.

Another limitation faced by this systematic review is the existence of studies that apply research tools, both screening and diagnostic, but do not use other tests as references, making comparison and static analysis impossible and, therefore, cannot be included in this article. Furthermore, it is possible to find professionals who incorrectly apply screening tests to establish a diagnosis.

### Future studies

Future studies should use an appropriate methodology to correct the biases that the authors find with a greater level of certainty in the evidence. It is necessary to have homogeneous and standardized screening methodologies before the diagnostic assessment becomes clear. Furthermore, the ages at which each tool will be applied must be strictly followed and the scores and questionnaires used must be standardized. With a uniform methodology, the results will be more accurate and reliable.

In this context, it is ideal to carry out new studies with an adequate, cross-sectional design, without pre-selection of patients to perform both screening and diagnosis, in order to reduce the risk of bias and increase the certainty of the evidence.

## Conclusion

It is mandatory to apply a screening test, the most recommended being the M-CHAT-R/F due to its sensitivity, specificity, likelihood ratio and post-test probability values. For diagnosis CARS and ADOS are the most recommended tools.

## Declaration of Competing Interest

The authors declare no conflicts of interest.

## Appendix Supplementary materials

Image, application 1
