# Supplementary material for: Screening and diagnostic tools for autism spectrum disorder: Systematic review and meta-analysis
Source: Clinics (Sao Paulo). 2024 Mar 14;79:100323. doi: 10.1016/j.clinsp.2023.100323 (PMC10951453; doi:10.1016/j.clinsp.2023.100323)
Supplement: Supplementary file 1 [file mmc1.docx]

**APPENDICIS**


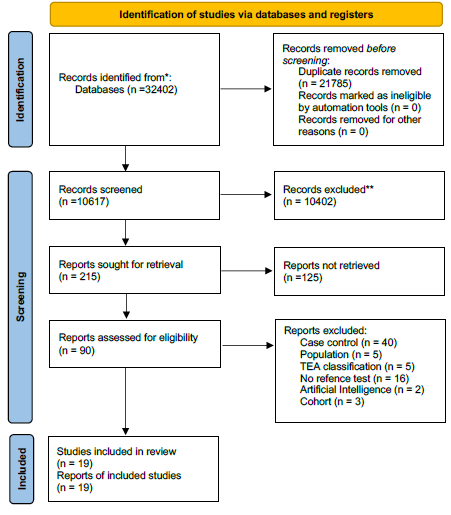


Figure 11 Flow diagram

From: Moher D, Liberati A, Tetzlaff J, Altman DG, The PRISMA Group (2009). Preferred Reporting Items for Systematic Reviews and Meta-Analyses: The PRISMA Statement. PLoS Med 6(7): e1000097. doi:10.1371/journal. pmed1000097

**Tabela 1** Description of screening and diagnosis tests applied in the studies included.

|  | **Tests** | | **Age range** | **Assessing** | **Average No. Items** | **Applied by** | **Scoring Method** | | **References** | |
| --- | --- | --- | --- | --- | --- | --- | --- | --- | --- | --- |
| **SCREENING** | **M-CHAT** | Modified Checklist for Autism in Toddlers | 18-24 months | Sensory abnormalities, motor abnormalities, social interchange, early joint attention/theory of mind, early language and communication. | 23 items | Parents | Each item is scored as "yes" or "no". The final score determining whether a child is at risk for autism or not. | 12 | |  |
|  | **M-CHAT-R/F** | Modified Checklist for Autism in Toddlers, Revised with Follow-Up | 16-30 months | Sensory abnormalities, motor abnormalities, social interchange, early joint attention/theory of mind, early language and communication. | 20 items | Parents + Professional (follow-up) | "yes" or "no" for classifications:  low risk (no further evaluation unless other risk factors are present)  medium risk (requires administration of the follow-up  high risk (immediate referral for evaluation and intervention). | 13 | |  |
|  | **Q-CHAT** | Quantitative Checklist for Autism in Toddlers | 18-30 months | Social communication, repetitive, stereotyped and sensory behaviours. | 25 items | Parents | Items are scored using a 5-point frequency scale ranging from 0 to 4, with higher scores indicating more autistic traits. | 44 | |  |
|  | **SCQ** | Social Communication Questionnaire | ≥4 years | Social, communication and repetitive behavior. | 40 items | Parents | Each item is scored as "yes" or "no" for lifetime or current form. The final score determining whether a child is at risk for autism or not. | 45 | |  |
|  | **RITA-T** | Rapid Interactive Screening Test for Autism in Toddlers | 18-36 months | Joint attention, social awareness, reaction to emotion, awareness of human agency, and object permanence. | 9 play-based scenarios | Professional | Maximum score is 30, with higher scores reflect more pronounced atypical behavior. The scores are categorized as follows: "low risk", "medium risk" (indicating a need for further investigation) and "high risk" for a formal diagnosis of ASD. | 46 | |  |
| **DIAGNOSTIC** | **ADOS** | Autism Diagnostic Observation Schedule | 5-12 years | Reciprocal social interaction, communication/language, stereotyped/restricted behaviors, and mood and nonspecific abnormal behaviors. | 8 Tasks | Professional | Scored on a 3-point scale from 0 to 2. The score classifies as diagnostic classifications of “autism,” “non-autism ASD,” and “non-spectrum”. | 42, 47 | |  |
|  | **ADI-R** | Autism Diagnostic Interview-Revised | ≥18 months | Communication, reciprocal social interaction, and restricted, repetitive behaviors and interests. | 93 items | Professional | Scored on a 4-point scale from 0 to 3. The score provides three diagnoses: "patient with autism", "autistic signs without the classic form of the disease" or "patient without autism". | 43 | |  |
|  | **ADOS-2** | Autism diagnostic observation schedule–2nd edition | ≥12 months | Social communication skills, restricted interests, and repetitive behaviors. | Tasks | Professional | Scored on a 3-point scale from 0 to 2. The score classifies as diagnostic classifications of “autism,” “non-autism ASD,” and “non-spectrum”. | 47 | |  |
|  | **CARS-2** | Childhood Autism Rating Scale, Second Edition | ≥2 years | Relationships with People, Imitation, Affect, Use of Body, Relation to Non-human Objects, Adaptation to Environmental Change, Visual Responsiveness, Auditory Responsiveness, Near Receptor Responsiveness, Anxiety Reaction, Verbal Communication, Nonverbal Communication, Activity Level, Intellectual Functioning, and the clinician’s general impression. | 15 items | Professional | Scored on a 4-point scale from 1 to 4. The score indicates "non-autism", "mild to moderate autism" or "severe autism". | 48 | |  |
|  | **SACS-R** | Social Attention and Communication Surveillance-Revised | ≥11-30 months | Social attention communication behaviours. | 43 items in total (12 month: 12 items; 18 month: 16 items; 24 month: 15 items) | Professional | Each item is classified in presenting with typical or atypical behaviour. Children with atypical behavioral presentation are considered at high likelihood for autism and referred for further assessment. | 49 | |  |
| **CRITERIAS** | **DSM-V** | Diagnostic and Statistical Manual of Mental Disorders - Fifth edition | - | Social communication and Restricted and Repetitive Behaviors. | 7 symptoms | Psychologist | Each symptom is scored as "absent", "present by history" or "currently present". The child is diagnosed with autism spectrum disorder if they meet the criteria. | 50 | |  |
|  | **DSM-IV** | Diagnostic and Statistical Manual of Mental Disorders - Fourth edition | - | Impairment in social interaction, impairments in communication and restricted, repetitive and stereotyped patterns of behavior, interests and activities. | 12 symptoms | Psychologist | Each symptom is scored as "present" or "absent". According to the criteria, the child can be diagnosed with "autistic disorder", "Asperger's disorder", or "pervasive developmental disorder - not otherwise specified" (PDD NOS). | 51 | |  |

A – RISK OF BIAS FROM INCLUDED STUDIES (QUADAS-2)

| **QUADAS-2 - SCREENING** | | | | |
| --- | --- | --- | --- | --- |
| **AUTHOR** | **PATIENT BIAS** | **INTREPTETION OF INDEX TEST HAVE INTRODUCED BIAS** | **REFERENCE STANDARD INTRODUCED BIAS** | **PATIENT FLOW HAVE INTRODUCED BIAS?** |
| **Jonsdottir SL** | LOW | LOW | HIGH | HIGH |
| **Mágan-Maganto M** | LOW | LOW | HIGH | LOW |
| **Kerub O** | LOW | LOW | HIGH | LOW |
| **Coelho-Medeiros ME** | LOW | LOW | HIGH | HIGH |
| **Srisinghasongkram P** | LOW | LOW | HIGH | LOW |
| **Kamio Y** | LOW | LOW | HIGH | HIGH |

**Table 2.** Risk of bias from screening studies.

| **QUADAS-2 - DIAGNOSTIC** | | | | |
| --- | --- | --- | --- | --- |
| **AUTHOR** | **PATIENT BIAS** | **INTREPTETION OF INDEX TEST HAVE INTRODUCED BIAS** | **REFERENCE STANDARD INTRODUCED BIAS** | **PATIENT FLOW HAVE INTRODUCED BIAS?** |
| **Barbaro J** | LOW | LOW | LOW | HIGH |
| **Vermeirsch J** | LOW | LOW | HIGH | LOW |
| **Mukherjee SB** | LOW | LOW | LOW | LOW |
| **Gulati S** | LOW | LOW | LOW | LOW |
| **Vats P** | LOW | LOW | LOW | LOW |
| **Mazurek MO** | LOW | HIGH | HIGH | HIGH |
| **Zander E** | LOW | LOW | LOW | LOW |
| **George B** | LOW | LOW | LOW | LOW |
| **Juneja M** | LOW | HIGH | HIGH | LOW |
| **Chlebowski C** | LOW | LOW | LOW | LOW |
| **Russell PS** | LOW | HIGH | HIGH | LOW |
| **Papanikolaou K** | LOW | LOW | LOW | LOW |
| **Risi S** | LOW | LOW | LOW | LOW |
| **Ventola P** | LOW | LOW | LOW | LOW |

**Table3** Risk of bias from diagnosis studies.

|  | **PMID** | **AUTHOR** | **YEAR** | **STUDY DESIGN** | **TEST** | **AGE**  **(MONTHS)** | **POPULATION** |
| --- | --- | --- | --- | --- | --- | --- | --- |
| **1** | **33945117** | Jonsdottir SL | 2022 | CROSS-SECTIONAL | M-CHAT-R/F | 31.66 (1.72) | 1586 |
| **2** | **30328577** | Magán-Maganto M | 2020 | CROSS-SECTIONAL | M-CHAT-R/F | 14-22  23-36 | 14-22: 3529  23-36: 3096 |
| **3** | **30099656** | Kerub O | 2018 | CROSS-SECTIONAL | M-CHAT/F | 21.30 (3.45) | 1591 |
| **4** | **31859732** | Coelho-Medeiros ME | 2019 | CROSS-SECTIONAL | M-CHAT-R/F | 16-30 | 120 |
| **5** | **27460003** | Srisinghasongkram P | 2016 | CROSS-SECTIONAL | M-CHAT-R/F | HR: 30 (26-35)  LR: 21 (18-30) | HR: 109  LR: 732 |
| **6** | **23740200** | Kamio Y | 2014 | CROSS-SECTIONAL | M-CHAT -R/F | 18 | 1851 |

**Table 4** Screening studies.

|  | **PMID** | **AUTHOR** | **YEAR** | **STUDY DESIGN** | **TEST** | **PATIENTS**  **(N)** | **AGE** | **POPULATION DETAIL** |
| --- | --- | --- | --- | --- | --- | --- | --- | --- |
| **1** | **35275169** | Barbaro J | 2022 | CROSS-SECTIONAL | SACS | 13511 | 11-30 months | Symptoms suggestive of autism |
| **2** | **32757085** | Vermeirsch J | 2021 | CROSS-SECTIONAL | ADOS - 2 | 55 | 3 years | Very preterm <32 weeks gestation. |
| **3** | **31724540** | Mukherjee SB | 2019 | CROSS-SECTIONAL | CARS2 | 500 | 2-5 years | Children with delay or regression of developmental milestones, abnormal language, or age inappropriate understanding, behavior, play and/or social interaction |
| **4** | **30865682** | Gulati S | 2019 | CROSS-SECTIONAL | ASD | 225 | 1-14 years | Suspect ASD when one of following features was present: 1. no babbling or ponting or other gesture by 12 months; 2. no single words by 16 months; 3. no 2-word spontaneous (not echolalic) phrases by 24 months; or 4. loss of language or social skills at any age. |
| **5** | **29978815** | Vats P | 2018 | CROSS-SECTIONAL | ASD | 118 | 2-9 years | Children with symptoms suggestive of ASD (delayed/deviant speech, poor eye contact, poor social skills, repetitive movements, delayed milestones, poor school performance, or hyperactivity). |
| **6** | **28620892** | Mazurek MO | 2017 | CROSS-SECTIONAL | DSM-5 | 439 | 2-17 years | Children referred for autism diagnostic evaluation |
| **8** | **24413849** | Zander E | 2015 | CROSS-SECTIONAL | ADI-R, ADOS-2 | 268 | 18-47 months | Unclear developmental concerns, for instance, language delay or global developmental delay, interaction difficulties and internalizing or externalizing behavior problems |
| **9** | **25428818** | George B | 2014 | CROSS-SECTIONAL | CARS | 200 | 2-6 years | Symptoms suggestive of autism |
| **10** | **24953575** | Juneja M | 2014 | CROSS-SECTIONAL | ASD, CARS | 154 | 2-9 years | Symptoms suggestive of autism |
| **11** | **20054630** | Chlebowski C | 2010 | CROSS-SECTIONAL | CARS | 2 years n=376;   4 years n=230 | 2 years (21-30 months)  4 years (42-66 months) | Childern referred for possible autism, who failed the modified checklist for autism in toddlers (M-CHAT) and a follow up thelephone interview (Robins et al. 2001) |
| **12** | **20490769** | Russell PS | 2010 | CROSS-SECTIONAL | CARS | 103 | 5.10 (2.20) | Children and adolescents suspected of having autism |
| **13** | **18752062** | Papanikolaou K | 2009 | CROSS-SECTIONAL | ADOS-G, ADI-R | 77 | 83 (44) months | Referred for clinical assessment to na outpatient PDD clinic. Referral sources included primary care clinical settings, independent profesionals, schools and parents |
| **14** | **16926617** | Risi S | 2006 | CROSS-SECTIONAL | ADI-R, ADOS | 1529 | 14 months -18 years | Patients with known developmental, cognitive, or behavioral diagnoses |
| **15** | 16897398 | Ventola | 2006 | CROSS-SECTIONAL | ADI-R, ADOS, CARS | 45 | 26 (16-30) months | Symptoms suggestive of autism |

**Table 5** Diagnosis studies.
